# Concluding Embryogenesis After Diaspora: Seed Germination in *Illicium Parviflorum*

**DOI:** 10.1093/icb/icad078

**Published:** 2023-06-22

**Authors:** Juan M Losada

**Affiliations:** Institute of Subtropical and Mediterranean Hortofruticulture La Mayora – CSIC – UMA. Avda. Dr. Wienberg s/n., Algarrobo-Costa, Málaga, 29750, Spain

## Abstract

Albuminous seeds, dispersed with a minimally developed embryo surrounded by nutrient storage tissue, are pervasive across extinct and extant early diverging angiosperm lineages. Typically, seed ontogenic studies have focused on the time between fertilization and seed release, but in albuminous seeds, embryogenesis is incomplete at the time of seed dispersal. Here, I studied the morphological and nutritional relationships between the embryo and the endosperm after seed dispersal in *Illicium parviflorum* (Austrobaileyales). Seeds of *I. parviflorum* germinate over a period of three months. Different stages during the germination process were anatomically evaluated using a combination of histochemistry and immunocytochemistry. At dispersal, the seeds of *Illicium* contain a tiny achlorophyllous embryo with minimal histological differentiation, surrounded by copious amounts of lipo-protein globules stored in the endosperm within cell walls rich in un-esterified pectins. Six weeks later, the embryo expanded and differentiated the vascular tissues before the emergence of the radicle through the seed coat, as the stored lipids and proteins coalesced within cells. Six weeks later, the cotyledons contained starch and complex lipids intracellularly, and accumulated low-esterified pectins in their cell walls. The proteolipid-rich albuminous seeds of *Illicium* exemplify how woody angiosperms of the Austrobaileyales, Amborellales, and many magnoliids release seeds with high-energy storage compounds that are reprocessed by embryos that complete development during germination. Seedlings of these lineages thrive in the understory of tropical environments, which match with the predicted habitats where angiosperms evolved.

## Introduction

More than half of the roughly 450 angiosperm families described to date store seed nutrients in a fully developed embryo, and hence are exalbuminous (Martin 1946). Most of these families belong to the eudicots (Forbis et al. 2002). Upon release, these embryos are typically mature to break the seed coat and establish an autonomous seedling (Linkies et al. 2010). However, in around half of extant monocots species (Sreenivasulu and Wobus 2013), in most magnoliids (Friis et al. 2015a,b, 2019), and in the basal grade of angiosperms that include *Amborella*, Nymphaeales, and Austrobaileyales (ANA), extant and extinct taxa release seeds with an immature minute embryo surrounded by substantial nutrient-storage tissue, which may be of maternal origin (i.e., perisperm) or a product of fertilization (i.e., endosperm); further and significant development of the embryo will occur after seed dispersal, although the degree of embryo differentiation varies across taxa (Grushvitzky 1967; Nikolaeva et al. 1985; Baskin and Baskin 1998; Friedman and Bachelier 2013; Povilus et al. 2015; Losada et al. 2017). During the early ontogenic steps of the embryo, the cotyledons are heterotrophic, and uptake nutrients from the surrounding nutritive tissue (reviewed in Povilus and Gehring 2022). In all albuminous seeds, following dispersal, the cotyledons within the seeds act as food transfer organs, and some never transition to nutrient storage (e.g., Nymphaeales: Friedman 2008; Friedman et al. 2012; Friedman and Bachelier 2013; Povilus et al. 2015, 2018; Lu and Magnani 2018; Dalziell et al. 2019; some members of the Annonaceae: de Vogel 1982). In most albuminous seeds with endosperm, the embryo uptakes nutrients from the endosperm until they emerge as photosynthetic, such as in *Amborella* and in the few Austrobaileyales studied to date (Chien et al. 2011; Fourcade et al. 2015; Fogliani et al. 2017; Losada et al. 2017). As a result, albuminous seeds with a massive endosperm often display prolonged temporal frames between seed dispersal from the maternal sporophyte and seedling establishment (Fogliani et al. 2017; Villegente et al. 2017), but the sequence of events that occur during this period are understudied. The albuminous seeds of the ANA grade offer an excellent system to better understand the largely unexplored spatiotemporal patterns of nutrient mobilization from the endosperm toward a maturing embryo after seed release (Losada et al. 2017).

**Fig. 1:**
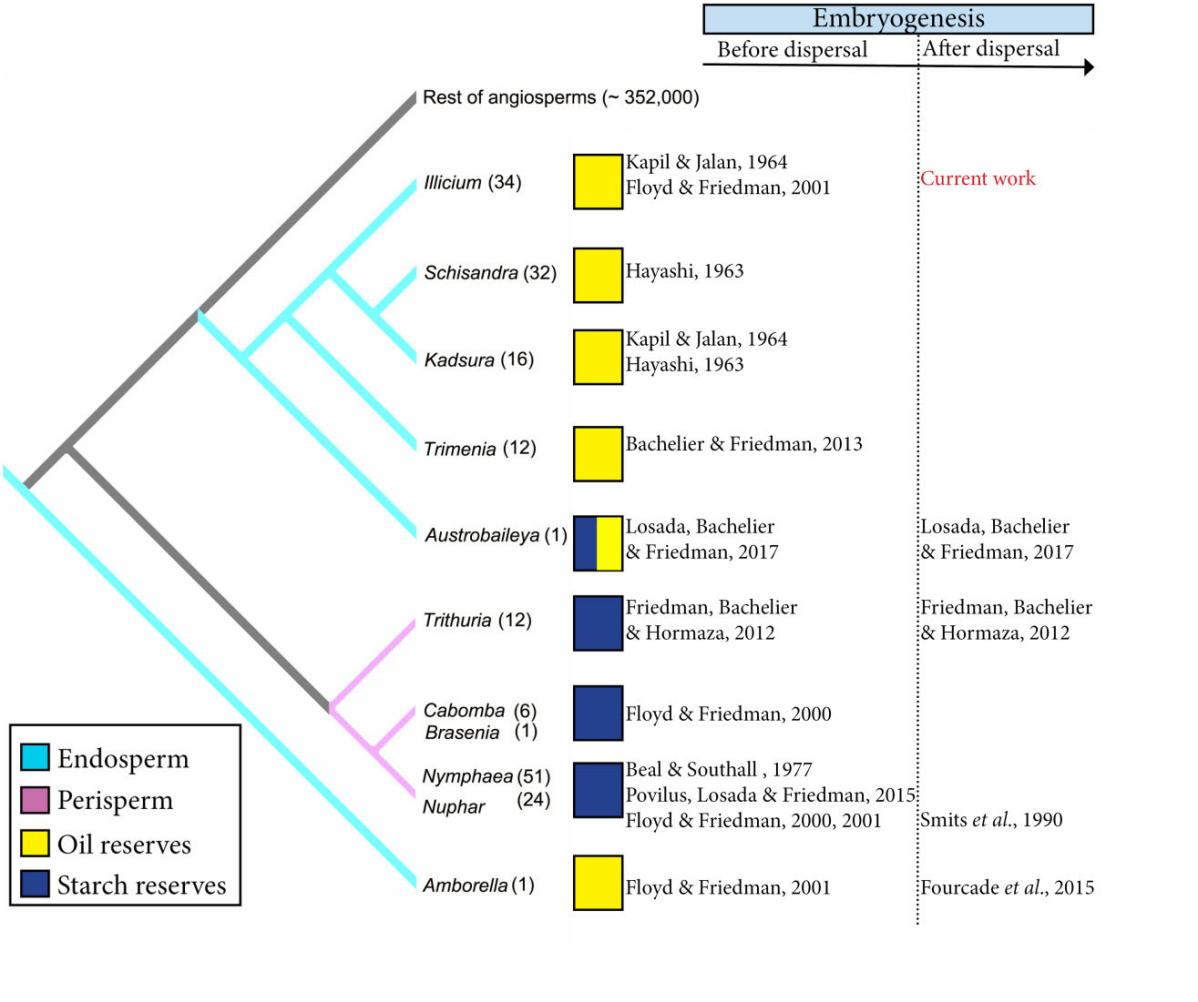
Phylogeny of the genera from the ANA grade of angiosperms with emphasis in the reserves contained within the storage tissues of albuminous seeds, either in the endosperm (light blue) or the perisperm (pink). Reserves accumulated within the storage tissues go from lipids and proteins (yellow) to starch and proteins (dark blue), and associated papers that reported their embryogeny, either before or after seed release. Phylogeny and data obtained from Stevens, 2001 onwards.

Albuminous seeds are likely a plesiomorphic condition of flowering plants (Forbis et al. 2002), and the nature of embryo-nourishing reserves accumulated in albuminous seeds can be divided into two basic types with a predominance of either starch or a combination of lipids and proteins. Rarely do these two types of reserves cooccur in the same storage tissues and accumulate either in the endosperm or in the perisperm (Friedman and Bachelier 2013). Intracellularly, starch storage occurs mainly within plastids, whereas processing of oil droplets involves a biochemical pathway that goes through the plastids, the endoplasmic reticulum, and eventually accumulates in the cytoplasm, and some steps are shared with proteins (Ohlrogge and Browse 1995; Herman and Larkins 1999). While many angiosperm species with albuminous seeds store starch in the endosperm or the perisperm at dispersal, for example, seeds of grasses (Zhao et al. 2018), woody members of the ANA grade lineages of flowering plants (Amborellales and Austrobailyeales), have seeds that are released with an endosperm loaded with lipo-protein bodies (Fig. 1). These characters have been reported in the families Amborellaceae (Amborellales, Floyd and Friedman 2001), Trimeniaceae (Austrobaileyales, Friedman and Bachelier 2013), or Schisandraceae (Austrobaileyales, Hayashi et al. 1963a, b; Kapil and Jalan 1964; Prakash 1998; Floyd and Friedman 2000, 2001). The only exception is the highly apomorphic monotypic Austrobaileyaceae, which produce massive seeds with a starchy endosperm, though combined with lipids and proteins (Endress 1980; Solntseva 1981; Yamada et al. 2003; Losada et al. 2017).

The earliest angiosperms were likely woody, and propagated their seeds in dark and humid environments, which correspond with tropical-like habitats (Feild and Arens 2007). Examples of these specialized habitats can be found in living members of the basal angiosperm grade, such as the shrubby *Amborella*, native to the tropical island of New Caledonia, or the lianoid *Austrobaileya*, specifically growing in the Australian tropical forest. The order Austrobaileyales is the sister lineage to all euangiosperms (Magnoliids, monocots, and eudicots), composed by three families of woody plants. Within the family Schisandraceae, the genus *Illicum*, containing around 40 species, is composed of shrubs native to shady tropical and warm temperate environments of the Asian and American continents, and the timing of diversification of the genus *Illicium* coincided with that between Nymphaeales and Chloranthales (Morris et al. 2007). Predispersal stages of seed development were previously reported (Floyd and Friedman 2001), but postdispersal information remains unstudied in most members of the Schisandraceae and the Austrobaileyales. To better understand the embryogenesis of albuminous seeds of the ANA grade and their evolution in angiosperms as a whole, we used the species *Illicium parviflorum* to evaluate the changes of both the embryo and the endosperm during seed germination.

## Materials and methods

### Plant material and seed collection

Plants of *I. parviflorum* were grown in plastic pots in the greenhouses of the Arnold Arboretum of Harvard University, using high porosity growing medium PRO-MIX (PremierTech Horticulture and Agriculture Group, Rivière-du-Loup, QC, Canada). Individual plants reached reproductive maturity under conditions of 25 ± 2°C and relative humidity (RH) of 75%, and due to their understory life habit, high sun exposure was avoided with a dark net between the greenhouse roof and the plants (average photosynthetic active radiation-PAR 100 µmol m^–2^ s^–1^).

**Fig. 2:**
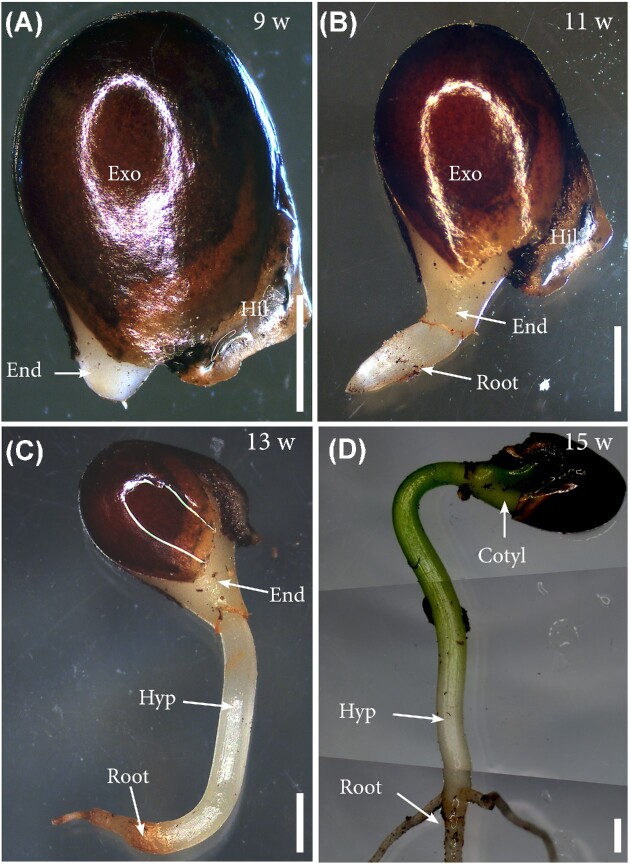
External morphology of *I. parviflorum* seeds during germination. (A) Germinated seed six weeks after dispersal/sowing (6w), showing breakage of the testa at the edge of the hilum, and protrusion of the micropylar endosperm. (B) Eleven weeks after dispersal (11w), the radicle breaks the endosperm cap. (C) The hypocotyl continues to elongate 13 weeks after dispersal (13w) but kept an achlorotic nature. (D) Fifteen weeks after dispersal (15w), the greening of the embryo was concomitant with the proliferation of cotyledons in an epigeal fashion (composite image). Cotyl, cotyledons; End, endosperm; Exo, exotesta; Hil, hilum; Hyp, hypocotyl. Scale bars: 2mm.

Mature plants flowered twice per year, and hand pollinations were performed at the anthesis stage using flowers from five different plants. While the fruit set was typically low (the average seed set per follicle was two out of six), from flowering to fruit dispersal about three-four months elapsed. Swollen fruits were collected prior to opening, and the seeds from the dehisced explosive follicles further collected for germination. Three seeds per pot were sown in PRO-MIX general soil (*n* = 200 seeds), keeping the soil moisture at field capacity. Samples of five seeds were collected at different temporal intervals from diaspora (3, 6, and 11 weeks), and up to 15 weeks when the first cotyledons emerged in an epigeal fashion (Fig. 2).

### Seed processing for histochemistry

Following seed collection, the exotesta of *I. parviflorum* seeds was removed with a scalpel, and then fixed in 4% acrolein (Polysciences, Inc.) in a modified PIPES buffer adjusted to pH 6.8 (50mM PIPES and 1mM MgSO_4_ from BDH, London, UK; 5mM EGTA from Research Organics, Inc., Cleveland, Ohio, USA). They were then dehydrated with an ethanol series up to 100% ethanol, infiltrated and embedded in glycol methacrylate resin (JB-4 Polysciences, Warrington, Pennsylvania, USA), and sectioned in 4μm–thick ribbons using glass knives mounted on a rotary microtome (Microm HM360 from Thermo Fisher Scientific, Waltham, Massachusetts, USA). Serial sections were mounted onto slides, stained with a periodic acid–Schiff's reaction (PAS) to detect insoluble carbohydrates, and counterstained with 0.01% aqueous toluidine blue (Feder and O'Brien 1968). Furthermore, sections were stained with 0.25% Naphtol Blue Black in 1% acetic acid (ABB) to stain proteins (Fisher 1968).

### Immunolocalization of low methyl esterified pectins

The germinating seeds of *I. parviflorum* were collected at the developmental stages described and, after removing the exotesta, fixed in 4% paraformaldehyde (Solís et al. 2008) in 0.1M Phosphate Buffer Saline (PBS). All fixed specimens were rinsed in PBS, and dehydrated through a graded series of acetone up to 100%. They were then embedded in Technovit 8100 (Electron Microscopy Sciences, USA), and sectioned at 4μm–thick ribbons as described above. Sections were placed on Superfrost slides, washed with PBS for 5 min, and pre incubated with 5% bovine serum albumin (BSA) for five more min. Then, we used the monoclonal antibody JIM5 (Knox 1997), which detects low methyl esterified pectin epitopes. The undiluted primary antibody was applied for 1 h, then washed in 1XPBS, and incubated again in the dark for 45 min with the 1/25 PBS diluted secondary antibody, anti-rat conjugated with Alexa 531 and 488 fluorochromes respectively (Thermo Fisher, Waltham, Massachusetts, USA). Following three washes in PBS, the sections were counterstained with calcofluor white to expose cell walls (Hughes and McCully 1975), and finally mounted in ProLong Gold Antifade reagent (Thermo Fisher) prior to microscopic observation.

### Digital imaging

Images of fresh seeds were taken using a Discovery AxioVision dissecting microscope (Carl Zeiss, Oberkochen, Germany). Photographs of microtome sections were taken using a combination of bright field and DIC, with the Zeiss Axio Imager Z2 microscope equipped with Zeiss High Resolution AxioCam digital cameras (Carl Zeiss, Oberkochen, Germany). Alexa 488 was visualized with a Zeiss LSM700 Confocal Microscope, equipped with an AxioCam 512 camera, illuminated with a fluorescence Lumencor SOLA-365-SE light, Chroma filter set 49002, excitation BP 470/40, beamsplitter T 495, emission BP 525/50. Figures involving whole seeds in all developmental stages are the result of photo merges of multiple individual images treated with the Adobe Creative Suite 5 (Adobe Systems, San Jose, California, USA).

## Results

### Postdispersal seed ontogeny of *I. parviflorum*

#### External morphology of I. parviflorum seeds during germination

Six weeks after seed sowing, the micropylar endosperm, pressed by the enlargement of the embryo, pushed and broke the hilum in *I. parviflorum* (Fig. 2A). Radicle emergence took place 3 weeks later (9 weeks after dispersal, Fig. 2B), without any dormancy requirements, but kept the two cotyledons growing into two white flat lobes at the expense of the endosperm inside the seed. As the hypocotyl elongated (Fig. 2C), the cotyledons continued their elongation inside the seed coat until just before they emerged from the soil, fully greened, in an epigeal fashion around fifteen weeks following dispersal (Fig. 2D).

#### Internal seed morpho-anatomy of I. parviflorum during germination

**Fig. 3:**
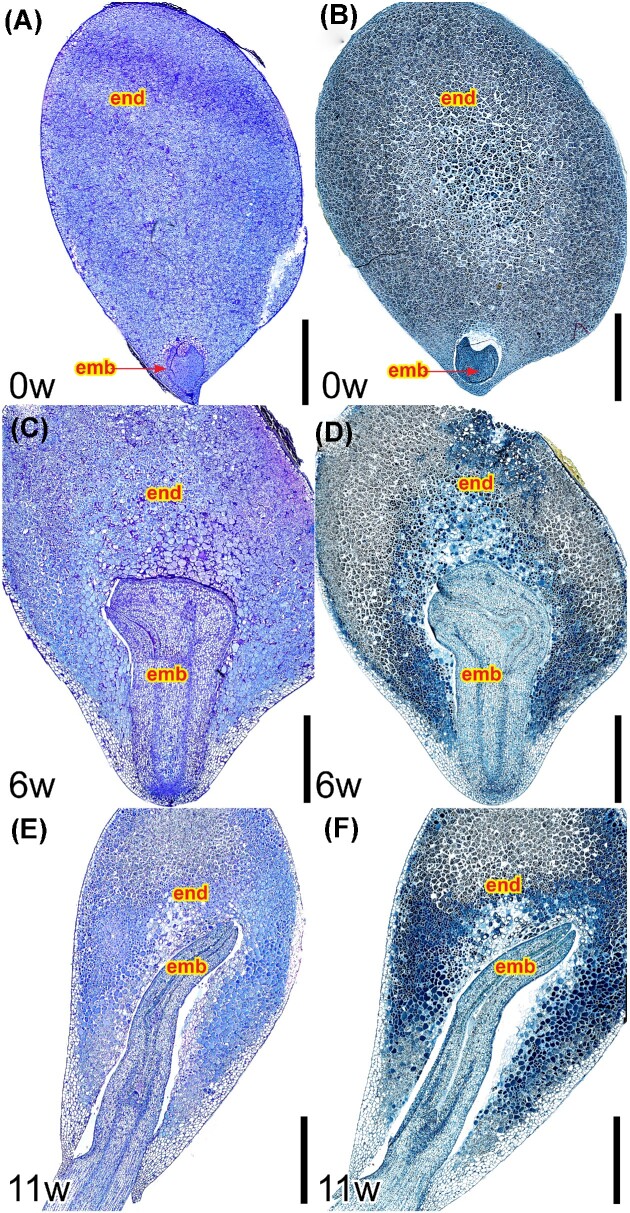
Internal morphology of *I. parviflorum* seeds during germination. (A) Fully expanded endosperm at the time of seed release (0w), combined with a tiny heart shaped embryo. B. Intense protein staining in both the endosperm and the embryo, except in the embryo surrounding area. (C) Six weeks later (6w), the embryo elongated within the seed, developed vascular bundles, and broke the exotesta, yet kept a layer of endosperm around the root tip. (D) At this stage, the endosperm displayed a more intense color in the areas around the expansion of the embryo. (E) Eleven weeks after seed release (11w), the cotyledons of the embryo occupied part of the endosperm area, and concomitantly, a big portion of the endosperm was depleted. F. Protein staining expanded to peripheral areas of the seed. Compositions of multiple images were obtained from 4 µm resin sections, stained with PAS for soluble carbohydrates (pink color), and counterstained with toluidine blue for general structure (blue color). Emb, embryo; end, endosperm. Scale bars: 2mm.

At the seed dispersal stage, a heart shaped embryo occupied a minimal part of the seed, displayed a protoderm, but no signs of protovascular tissue, and the majority of the seed was occupied by the endosperm, devoid of starch reserves (Fig. 3A), but stained intensely for proteins and lipids (Fig. 3B). Six weeks later, the embryo expanded inside the seed, pressing both the micropylar and chalazal endosperm area (Fig. 3C), where the staining for proteins was more intense around the embryo (Fig. 3D), while the vascular bundles differentiated. Eleven weeks after dispersal, the root exited the seed, the embryo elongated the hypocotyl and cotyledons occupied a larger area of the endosperm (Fig. 3E), which continued to stain intensely for proteins toward the seed periphery (Fig. 3F).

#### Polysaccharides in the embryonic tissues

**Fig. 4:**
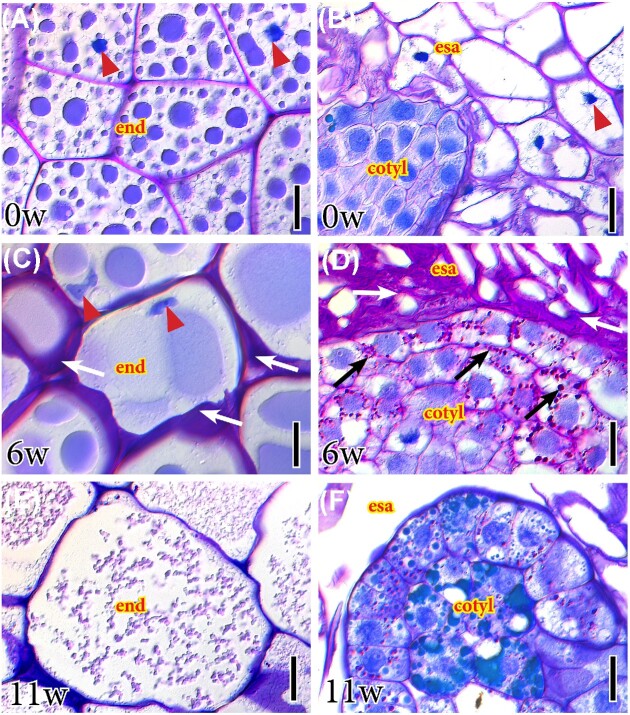
Polysaccharides in the endosperm and the embryo during seed germination of *I. parviflorum*. (A) At the time of seed dispersal (0w), the endosperm is composed of nucleated storage cells (red arrowheads). (B) In contrast, the area of the endosperm surrounding the embryo was devoid of cytoplasmic material, yet keeping small nuclei (red arrowhead). (C) Six weeks after dispersal (6w), the cellular contents of the endosperm coalesced, and pushed the nuclei against cell walls (red arrowheads). (D) The embryo surrounding area was a tightly packed mass of cell walls (white arrows), as starch accumulated in the developing cotyledons (black arrows). (E) Eleven weeks after seed release, the contents of the endosperm cells disintegrated and cell walls thinned down. F. The embryo differentiated the epidermal and subepidermal tissues. 4µm resin sections stained with PAS for insoluble polysaccharides (pink), counterstained with toluidine blue for general structure (blue color). Cotyl, cotyledon; emb, embryo; end, endosperm; esa, embryo-surrounding area. Scale bars: 20µm.

Detailed evaluations of the endosperm and embryo tissues revealed that, at dispersal, the endosperm cells contained numerous cytoplasmic globular bodies and conserved their nuclei (Fig. 4A). In contrast, the area around the embryo was devoid of cell contents, but maintained the integrity of the cell walls (Fig. 4B). Six weeks later, the endosperm cell contents coalesced, pushing the nuclei toward the edges of the cells, and the intercellular spaces enlarged, where insoluble polysaccharides accumulated (Fig. 4C). The embryo surrounding area displayed a high concentration of sugars, with compressed cell walls, as cotyledons accumulated starch (Fig. 4D). At later germination stages, the endosperm cell contents disintegrated, their nuclei were no longer detectable, and the cell walls thinned down (Fig. 4E). Concomitantly, the cotyledons displayed differentiation of two external dermal layers, and the internal one accumulated tannins (Fig. 4F).

#### Lipids and proteins in the embryonic tissues

**Fig. 5:**
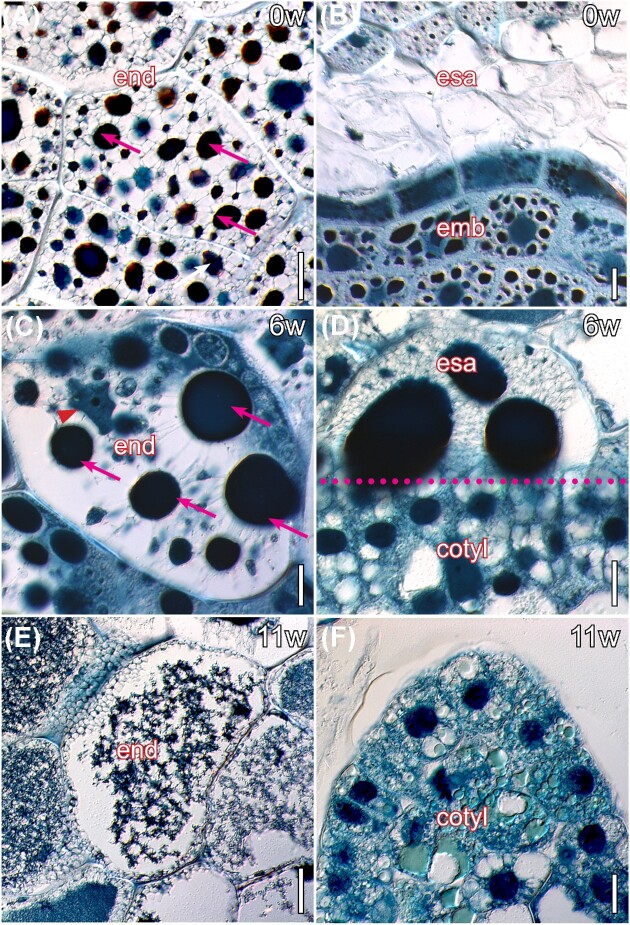
Proteins in the endosperm and the embryo during seed germination of *I. parviflorum*. (A) At the time of seed dispersal (0w), the endosperm is composed of condensed cells with protein bodies (arrows). (B) While the embryo surrounding area of the endosperm is devoid of proteins, the cotyledons contained numerous small protein bodies. (C) Six weeks after dispersal (6w), larger protein bodies within endosperm cells suggested coalescence, contained in nucleated cells (red arrowhead). (D) The embryo surrounding area showed contact of endosperm proteins with the expanding embryo (the dotted line depicts the boundary between the endosperm and the embryo). (E) Eleven weeks after seed release, proteins broke down into small pieces that traversed cell walls. (F) In the differentiated embryo, proteins concentrated in the nuclei, and the sub-epidermal tissues displayed cell compounds with birefringence (arrows). A total of 4µm resin sections stained with ABB for general proteins (dark blue) imaged with differential interface contrast (DIC). Cotyl, cotyledon; emb, embryo; end, endosperm; esa, embryo-surrounding area. Scale bars: 20µm.

The absence of protein stain in the cell walls contrasted with intense staining of the globular contents of the endosperm cells (Fig. 5A). Proteins were not present in the embryo surrounding area (Fig. 5B). Six weeks after sowing, proteins from the endosperm coalesced onto larger vesicles, and nuclei were still visible (Fig. 5C). The expanding embryo contacted directly the coalesced proteins (Fig. 5D). At later stages, the proteins broke down into smaller fragments that stained across cell walls (Fig. 5E). The differentiated cotyledons showed most protein staining in their cell nuclei, more slightly in the cytoplasm, and compounds that reacted faintly for proteins accumulated under the epidermis (Fig. 5F).

**Fig. 6:**
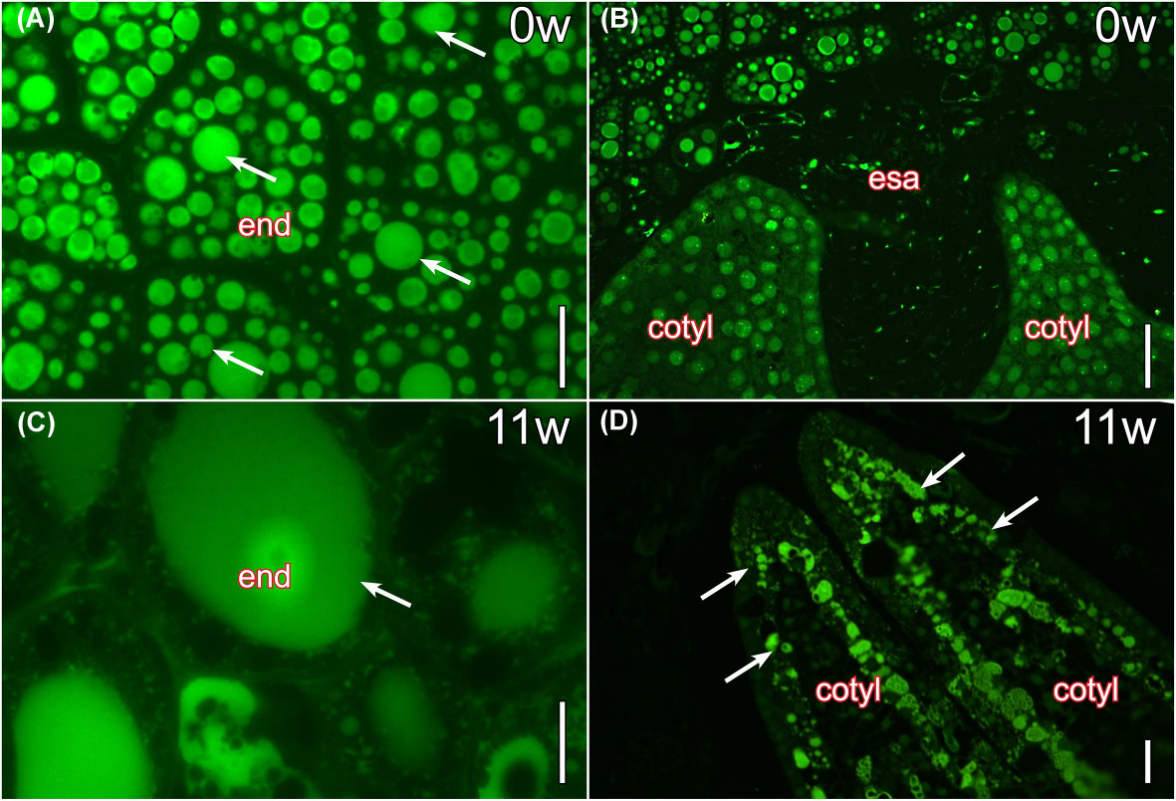
Lipids in the endosperm and the embryo during seed germination of *I. parviflorum*. (A) The endosperm was filled with lipid globules at the time of seed release. (B) The embryo surrounding area contained some remnants of lipids compared with other areas of the endosperm. (C) Lipids within endosperm cells coalesced and filled most of the areas in the endosperm cells. (D) The cotyledons differentiated a subdermal layer that reacted for lipids at germination. A total of 4µm resin sections stained with auramine O for lipids (green). Cotyl, cotyledon; end, endosperm; esa, embryo-surrounding area. Scale bars: A, C, 20µm; B, D, 100µm.

The vesicles of the endosperm cells at seed dispersal further reacted for lipids, except in the cell walls (Fig. 6A). Smaller lipidic vesicles were present in the embryo surrounding area (Fig. 6B). Similar to proteins, the lipids of the endosperm coalesced prior to seedling emergence (Fig. 6C), and the subdermal layer of the cotyledons accumulated substances that further reacted for lipids (Fig. 6D).

#### Pectin epitopes in the cell walls of embryonic tissues

**Fig. 7:**
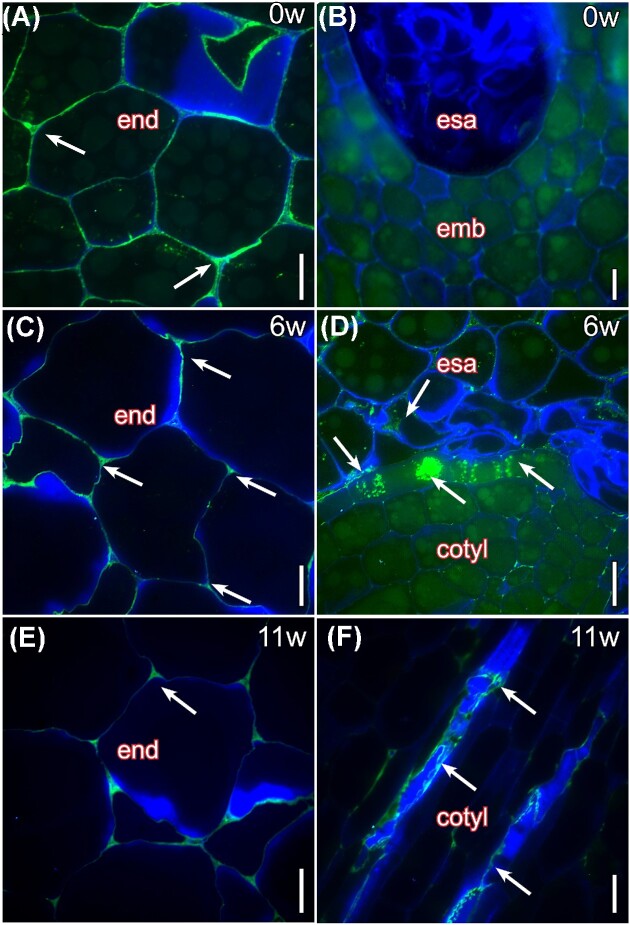
Immunolocalization of low methyl-esterfied pectins recognized by the JIM5 mAb in the endosperm and the embryo of *I. parviflorum* during seed germination. (A) At the time of dispersal (0w), the endosperm cell walls contained unesterified pectin epitopes (white arrows). (B) In contrast, the developing embryo did not accumulate pectins that conjugate with JIM5 in the cell walls. (C) Six weeks after dispersal (6w), the pectin epitopes hyper accumulated in the apoplastic spaces of the endosperm (white arrows). (D) In the embryo surrounding area, as endosperm cells collapsed, pectin epitopes were released to the apoplastic space, and localized in the epidermal layers of the developing embryo (white arrows). (E) Twelve weeks after diaspora (12w), pectin epitopes vanished from the endosperm cell walls (white arrow). (F) In the embryo, however, the cell walls of the cotyledons accumulated high amounts of pectins (white arrows), most remarkably in the proto-vasculature. *Illicium parviflorum* seeds immunolocalized with JIM5 monoclonal antibody linked to a FITC-labelled secondary antibody (green), counterstained with calcofluor white for cellulose (blue). Cotyl, cotyledon; end, endosperm; emb, embryo; esa, embryo-surrounding area. Scale bars: 20µm.

Low methyl esterified pectins pervaded the cell walls of the mature endosperm in *I. parviflorum* at the seed dispersal stage (Fig. 7A). In contrast, the immature embryonic cell walls contain little of these pectins (Fig. 7B). During the initial stages of embryo elongation, pectins of the endosperm cell walls hyperaccumulated in the apoplastic spaces (Fig. 7C), including the collapsed cells around the embryo surrounding area, and were concomitantly detected in the epidermal tissues of the embryo (Fig. 7D). At later stages of seed germination, pectins vanished from the intercellular spaces of the endosperm (Fig. 7E), but the neoformed walls of the embryo accumulated pectins, especially in the protovasculature (Fig. 7F).

## Discussion

The complete embryogenesis of albuminous seeds of the basal angiosperm grade genera such as *Illicium* contributes to a better reconstruction of the developmental pathways of embryonic tissues in the earliest angiosperm lineages.

### Delayed embryo maturation in albuminous seeds of the ANA grade

Embryogenesis of *I. parviflorum* is completed between three and four months after seed dispersal, when disconnected from the mother plant (see also Thien et al. 1983; Olsen and Ruter 2001). The temporal frame that elapses between seed release and testa break takes more than 2 months in woody members of the ANA grade. Thus, *Amborella* takes about 3 months for testa break (Fourcade et al. 2015), and within Austrobaileyales, this temporal frame varies from 2 months of studied species of *Schisandra* (Chien et al. 2011; Hayashi 1963a,b), to the unusually prolonged 12 months of *Austrobaileya* (Losada et al. 2017). In contrast, the seeds of the aquatic herbs Nymphaelales typically display periods of germination of days in absence of physiological dormancy (Beal and Southall 1977; Friedman et al. 2012; Povilus et al. 2015). Causes behind delayed rooting in albuminous seeds of the ANA grade remain conjectural, but are unlikely related to the dispersal syndrome, given that living species display a wide range of seed dispersal mechanisms, from zoochory (Austrobaileya (Endress 1980), hydrochory (all Nymphaeales, see Povilus et al. 2015) or anemochory (p.e. the explosive fruits of *Illicium*: Romanov et al. 2013). In contrast, delay in seedling establishment, or morphological dormancy, is considered an ancestral type of dormancy in angiosperms (Willis et al. 2014), related with the release of seeds with rudimentary embryos (Forbis et al. 2002), a pervasive feature across all members of the basal angiosperm grade and most magnoliids (Friedman 2006; Baskin and Baskin 2007; Friedman et al. 2012; Povilus et al. 2015 and refs).

The maturity (i.e., ontogenic stage) of the embryo at the time of seed dispersal has diversified significantly across angiosperms (Finch-Savage and Leubner-Metzger 2006; Linkies et al. 2010; Willis et al. 2014), associated with heterochronic shifts in the developmental program of the embryo (Forbis et al. 2002), sometimes enforced by the environment. For example, plants that live in extreme saline environments, such as mangroves or aquatic marine plants, release embryos “overdeveloped” (i.e., as seedlings), termed as viviparous plants, a strategy to ensure survival (Sinclair et al. 2015). The majority of eudicot species disperse a fully phostosyntetic embryo contained within the seed (exalbuminous; Forbis et al. 2002), and, unlike seeds of the ANA grade, evolved the ability to sense environmental clues, thus expanding the possibility to establish seedlings immediately after dispersal (non-dormant), or waiting until the conditions are favorable (dormant), such as in the model plant *Arabidopsis* (O´Neil et al. 2019; Doll et al. 2020). Although this degree of specialization has been observed in albuminous seeds of grasses (Leroux et al. 2014), the albuminous seeds of early diverging lineages, restricted to scenarios of low light combined with high humidity, likely lacked this independence, resulting in slower germination speeds. Germination speed along with seed longevity implies a bet-hedging strategy across angiosperms, given that acceleration of the embryo developmental program might have resulted in the release of more competitive seeds with enhanced resilience to extreme environments (drier, cooler, or higher light exposure, among others) (Finch-Savage and Footitt 2017; Fernandez-Pascual et al. 2019).

### Completion of embryogenesis during germination of albuminous seeds

During the initial stages of *I. parviflorum* seed germination, prior to radicle emergence, the sequence of events in the endosperm involves coalescence of intracellular proteins and lipids, and their release to the apoplastic space around the elongating embryo. The heterotrophic character of the cotyledons is visually displayed by their achlorophyllous nature together with the absence of a defined cuticle on their surface. Mobilization of nutrients stored in the endosperm is concomitant with the biosynthesis of starch in the cotyledons and the gradual differentiation of tissues involved in transport, such as the vascular bundles. This stage is common to all albuminous seeds, but while some taxa have cotyledons as haustorial-foodstoring organs, such as all Nymphaeales (Friedman 2008; Friedman et al. 2012; Friedman and Bachelier 2013; Povilus et al. 2015, 2018; Lu and Magnani 2018; Dalziell et al. 2019), and some members of the Annonaceae family (Magnoliales, de Vogel 1982), termed as cryptocotylar seed germination, most albuminous seeds transition to greening and their cotyledons emerge, named phanerocotylar seed germination (Finneseth et al. 1998). In germinating *Illicium* seeds, radicle emergence occurs before the biosynthesis of protective materials such as suberin-like compounds in the sub-epidermal tissues, and the accumulation of pectins in the cell walls of the cotyledons while greening occurs. The putative uptake of pectins from the endosperm cell walls by the developing cotyledons has been reported in model species with albuminous seeds, but so far, there are no reports of their localization across members of the ANA grade. Most works on the pectins from the endosperm come from predispersal stages of albuminous seeds of herbaceous plants such as grasses [*Oryza* (Palmer et al. 2015), *Hordeum* (Selvig et al. 1986), *Triticum* (Chateigner-Boutin et al. 2014), or *Brachypodium* (Guillon et al. 2011)], or exalbuminous seeds such as *Arabidopsis* (Cruz-Valderrama et al. 2018) and *Nicotiana* (Lee et al. 2013). These works pointed to a nutritional role of the pectins from the endosperm, supposedly reprocessed into new embryonic cell walls (Lu and Magnani 2018; Steinbrecher and Leubner-Mezer 2018). Yet, a handful of works have explored the presence of pectins in the embryogenesis of woody organisms with albuminous seeds, such as *Quercus* (Lopes et al. 2016; Costa et al. 2019). Our work suggests a tropic role of pectins during the transition from heterotrophy to autotrophy (i.e., greening and emergence of cotyledons), which occurs when seeds are separated from the mother plant.

### Oily endosperms in the albuminous seeds of Austrobaileyales

In *I. parviflorum*, the compounds stored within cells of the endosperm are lipids and proteins, and we noticed that this association is true for most woody members of Austrobaileyales (Humphrey 1896; Floyd and Friedman 2000, 2001; Friedman and Bachelier 2013), including the monotypic Austrobaileyaceae, which displayed not only lipids and proteins in the endosperm, but also starch deposits (Slontseva 1981; Losada et al. 2017). In species with a starchy perisperm as the main storage tissue, lipids and proteins were detected in their highly reduced endosperm, such as in *Nuphar* (Floyd and Friedman 2001). Previous evaluation of lipids in the seeds of a range of angiosperm species (not including the ANA grade), revealed a tight association between higher seed oil content and both woodiness and adaptation to darkness (Levin 1974; Sanyal and Decoq 2016). The genus *llicium* and other members of the Austrobaileyales are composed of species that thrive in the understory, adjusting well to this correlation. In addition, the earliest angiosperms were likely woody and evolved in stable environments with low light and high humidity (Finkelstein and Grubb, 2002; Feild and Arens 2007), pointing to albuminous-oily seeds as common in these scenarios (Floyd and Friedman 2000).

The question remains on what is the benefit, if any, of storing lipids and proteins. While proteins are universally stored within seeds, and provide the materials for the enzymatic machinery that catalyzes biosynthetic pathways (Altshul et al. 1966; Purkrtova et al. 2008; Yan et al., 2014), lipids and carbohydrates provide the energy, typically in the form of ATP (Quettier and Eastmond 2009; Bates et al. 2013; Ohlrogge and Browse 1995). However, lipids appear as more efficient packing compounds, given that oils provide a higher energy per unit mass compared with carbohydrates (Levin 1974). Additionally, lipids would prevent the dehydration of seeds more efficiently than starch (Westgate 1994). Starch, on the other hand, appears to be more readily available, and this could be related with higher germination rates, such as those observed in some herbaceous plants (Xu et al. 2014; Zhao et al., 2018). The hydrophobicity of the lipids contained within the endosperm of albuminous seeds might increase seed longevity under scenarios of high humidity (Ellis et al. 1989), whereas pectins from the endosperm cell walls might play the role on seed humectation during embryo activation. While nutrient mobilization toward a growing embryo has been thoroughly documented in herbaceous lineages of the ANA grade with starchy albuminous seeds (Nymphaeales: Friedman et al. 2012; Povilus et al. 2015), more works are needed for a thorough reconstruction of the seed germination trajectories in albuminous seeds of woody lineages (Losada et al. 2017; Fig. 1).

## Conclusion

Seed dispersal affects species distribution and community assembly, but does not mean the beginning of autonomous life in all angiosperms (Ingram 2020). While the essential toolkit for an independent life is built in the embryos of exalbuminous plants at the time of seed release, in albuminous plants from the basal grade of angiosperms (and likely other taxa with underdeveloped embryos at seed maturity), complete tissue differentiation is fully dependent on endosperm (Amborellales and Austrobaileyales) or perisperm (Nymphaeales) provisioning. Surmounting the boundaries of a seed is a major step in the life cycle of a new sporophyte, but during the initial steps of angiosperm evolution, embryo autonomy was achieved separated from the mother plant aided by a long-lived endosperm. The seeds of *I. parviflorum* illustrate how living members of early branches of flowering plants contribute to a better understanding the tremendous diversification of seed morpho-physiological traits across angiosperms from ancestral immature embryos.

## Funding

This work has been partially funded by a Putnam Scholarship from the Arnold Arboretum of Harvard University, and the Spanish Ministry of Science and Innovation, through the Agencia Estatal de Investigación, with a reference PID2021-129074OB-I00.

## Data Availability

Data will be available upon request to the author.
